# Management of jugular tympanic paraganglioma: a case report

**DOI:** 10.11604/pamj.2022.43.166.29457

**Published:** 2022-12-02

**Authors:** Mehdi Ferjaoui, Naourez Kolsi, Wiem Boughzala, Ons Kharrat, Rachida Bouatay, Khaled Harrathi, Amel Elkorbi, Jamel Koubaa

**Affiliations:** 1 Ear, Nose, Throat Department of Fattouma Bourguiba, University Hospital of Monastir, Resaerch Laboratory LR18Sp08, Monastir, Tunisia

**Keywords:** Jugular tympanic paraganglioma, tinnitus, medical imaging, surgery, case report

## Abstract

Paragangliomas could be localized from the skull base to the pelvic floor. Tympanic localization represents the most common benign tumor of the middle ear. Diagnosis is based on clinical signs with a great contribution of radiology. A 40-year-old male presented with isolated tinnitus of the right ear evolving for 18 months. Examination revealed a red bulging right-sided tympanic membrane and a conductive hearing loss. Tomodensitometry and Magnetic resonance imagery showed findings in favor of a right jugular tympanic paraganglioma. The tumor was classified type B according to FISCH classification. The patient underwent surgery consisting in tympanotomy using a retro auricular access route. The postoperative course was uneventful. There was no recurrence during the one-year follow-up. Jugular tympanic paraganglioma diagnosis is guided by a combination of epidemiological, clinical and radiological features. Treatment is still not consensual, but surgery still have its indications in localized forms of head and neck paragangliomas (HNP´s).

## Introduction

Paragangliomas could be localized from the skull base to the pelvic floor. They are embryologically derived from the neural crest and arise from paraganglia cells within a highly vascular environment. They are classified by their location. Head and neck paragangliomas (HNPs) originate from the parasympathetic tissue, with the carotid body, vagal body and jugular tympanic region are the most common locations [1]. Tympanic paraganglioma represents the most common benign tumor of the middle ear. Diagnosis is based on clinical signs with a great contribution of imagery technics. Treatment is not consensual, but surgery still has its place in some forms of HNPs.

## Patient and observation

We report the case of a jugular tympanic paraganglioma, diagnosed and treated in our ENT department in 2019.

**Patient information**: a forty-year-old male, smoker, presented in consultation with gradually increasing, unilateral, right tinnitus, evolving for 18 months, associated with hear loss. Tinnitus was swishing as reported by the patient, synchronous with pulse, and responsible of an important discomfort, causing concentration difficulties and insomnia. The patient did not report any otorrhea, otalgia or vertigo. He did not report any sign for the left ear. His past medical history was normal for any cerebrovascular accident, migraine, headaches, head and neck trauma, or radiation exposure. He had no family history of paragangliomas nor multiple endocrine neoplasia. He did not any past interventions.

**Figure 1:**
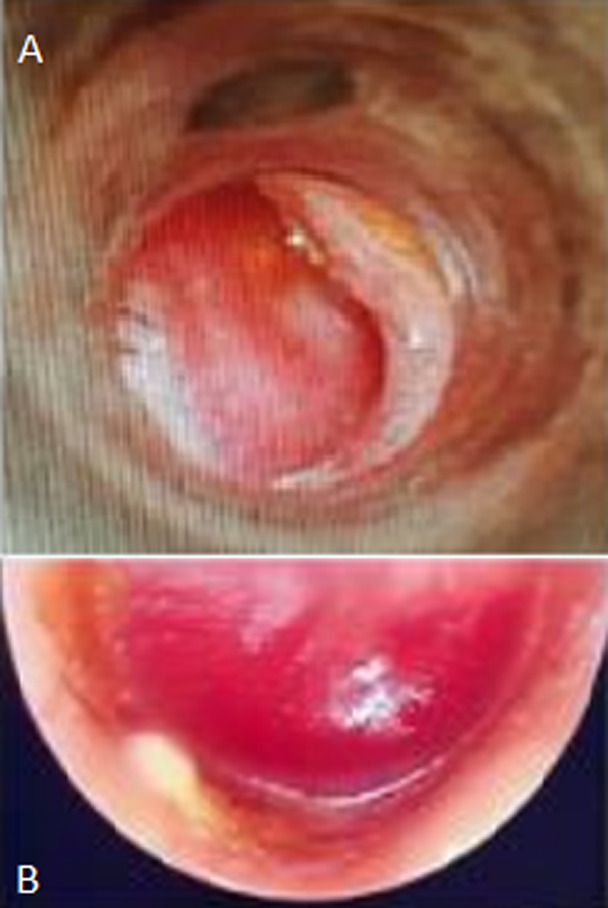
the bulging of the tympanic membrane produced by a glomus tumor: A) otoscopic examination; B) endoscopic examination showing a red pulsatile swelling behind the lower part of the right ear-drum

**Clinical findings**: otoscopic examination, using a rigid endoscope 0° (Figure 1), revealed a red pulsatile swelling behind the lower part of the right ear-drum. The Weber test was lateralized to the pathologic ear, and the Rinne test was negative. Carotid manual pressure temporarily stopped the tinnitus. The rest of the physical examination showed no neurological deficits, particularly no defects corresponding to one or more of IX^th^, X^th^, XI^th^ and XII^th^ cranial nerves.

**Timeline of current episode**: the symptoms had been evolving for 18 months with a gradual exacerbation.

**Figure 2:**
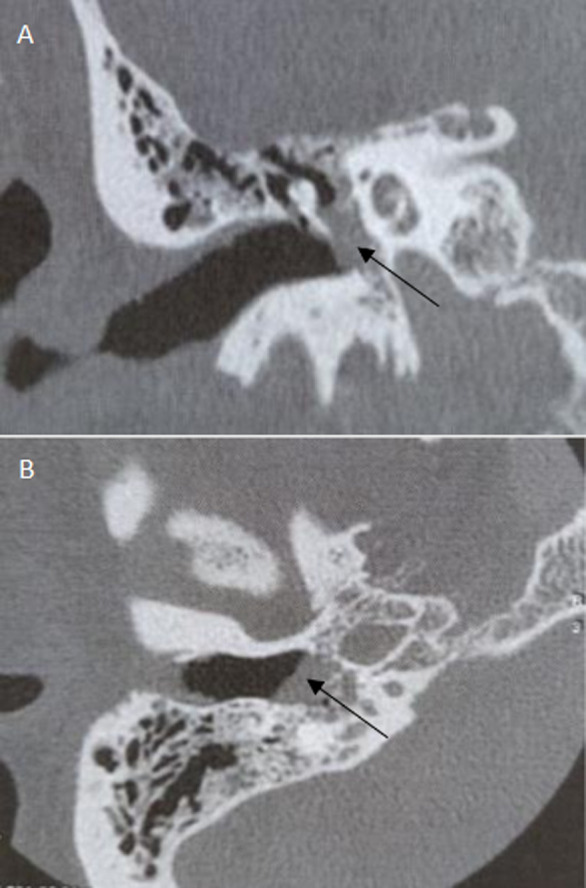
temporal bones computerized tomography (CT), coronal (A) and axial (B) sections, without contrast, showing a nonspecific, well-defined, 7 mm right nodular mass of soft tissue, arising from the hypotympanum and overlying the cochlear promontory

**Figure 3:**
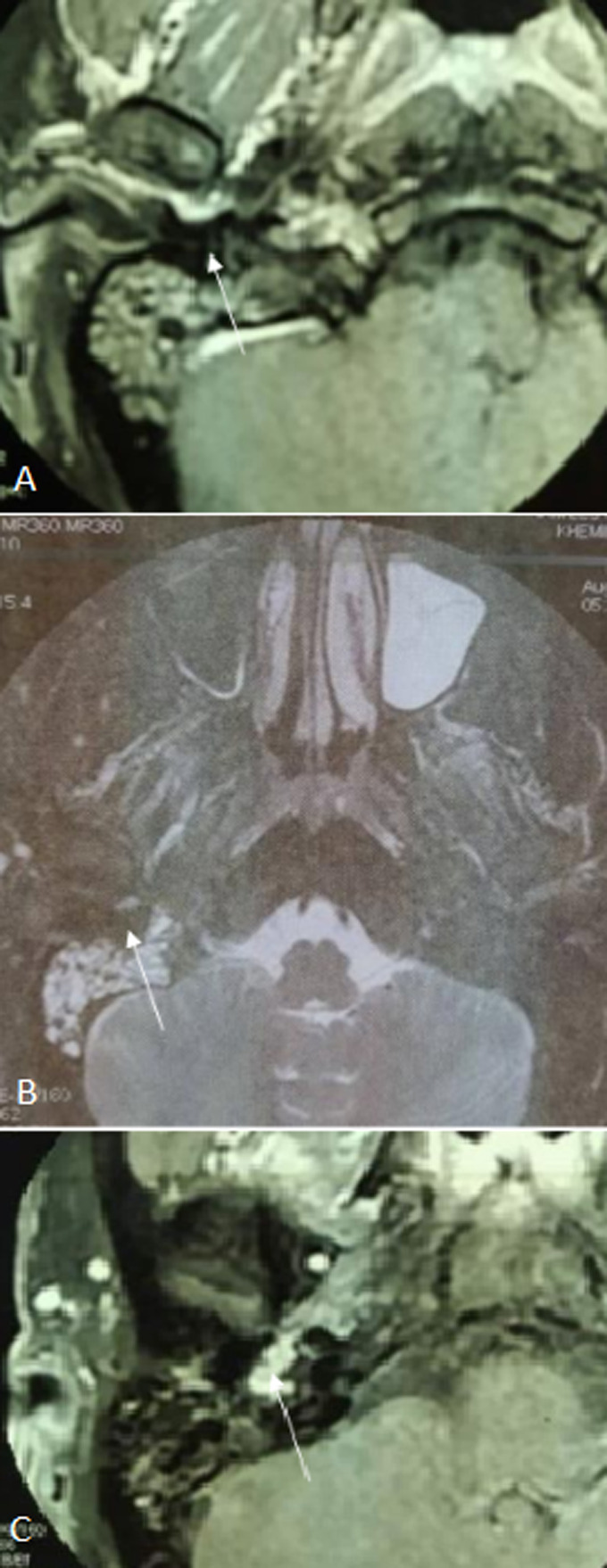
MRI showing a 15 mm mass of the right tympanic cavity, with a “salt and pepper” pattern of hypo intensity and hyper intensity on T1-weighted images (A), relatively higher in intensity on T2-weighted images (B), and strongly enhanced after gadolinium injection (C)

**Figure 4:**
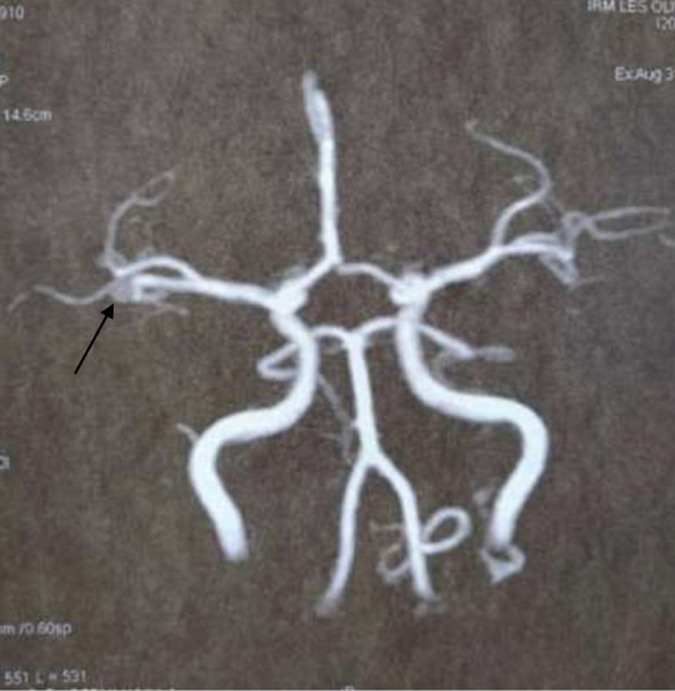
angio-MRI sequences showing a hyper vascular lesion

**Diagnostic assessment**: pure-tone audiometry showed a right conductive hearing loss of 30dB. No abnormalities were detected on the left side. Temporal bones Computerized tomography (CT), axial and coronal sections, without contrast, showed a nonspecific, well-defined, 7 mm right nodular mass of soft tissue, arising from the hypotympanum and overlying the cochlear promontory (Figure 2). It reaches the oval and the round foramina without any bone erosion. The ossicular chain, the facial nerve canal and the inner ear were intact. MRI showed a 15 mm mass of the right tympanic cavity, with a “salt and pepper” pattern of hypo intensity and hyper intensity on T1-weighted images, relatively higher in intensity on T2-weighted images, and strongly enhanced after gadolinium injection (Figure 3). On diffusion-weighted images, it appeared in hypersignal. The lesion reaches the tympanic membrane and expands through the aditus ad antrum into the mastoid antrum. These findings appeared to be consistent with jugular tympanic paraganglioma. There were no signs of invasion of the skull base. MRI was completed with angio-MRI sequences showing a hyper vascular lesion corresponding to the glomus tympanicum tumor (Figure 4).

**Diagnosis**: considering the radiological examination, the paraganglioma was classified type B according to FISH classification of jugular tympanic paragangliomas (Table 1).

**Table 1:** the FISCH classification of jugulotympanic paragangliomas

Class	Characteristics
A	Limited to mesotympanum (glomus tympanicum)
B	Limited to hypotympanum, mesotympanum, and mastoid with/without erosion of the jugular bulb (glomus hypotympanicum)
C	Involvement and destruction of infra-labyrinthine and apical compartments
C1	No invasion of vertical carotid canal; destruction of the jugular foramen
C2	Invasion of vertical carotid canal between foramen and bend
C3	Invasion along horizontal carotid canal
C4	Invasion of foramen lacerum and along carotid canal into cavernous sinus
D	Intracranial extension
De1	≤2 cm dural displacement
De2	>2 cm dural displacement
Di1	≤2 cm intradural extension
Di2	>2 cm intradural extension
Di3	Inoperable intracranial invasion

**Prognostic characteristics**: the Type B paraganglioma is still localized to middle ear, surgery could be complete and successful for our case.

**Figure 5:**
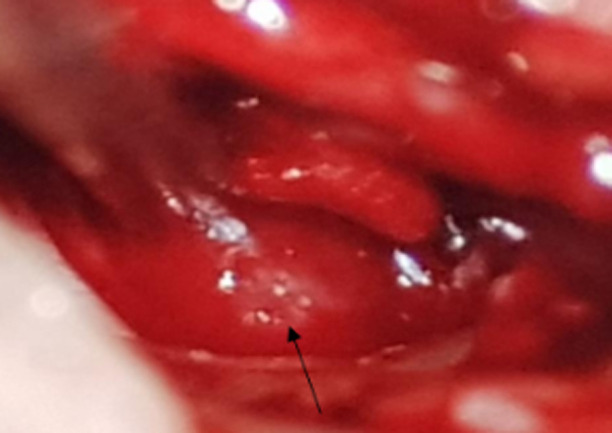
per-operative view: the tumor was hyper vascular, easily bleeding, emerging from the hypotympanum, filling the tympanic cavity and occluding the Eustachian tube

**Figure 6:**
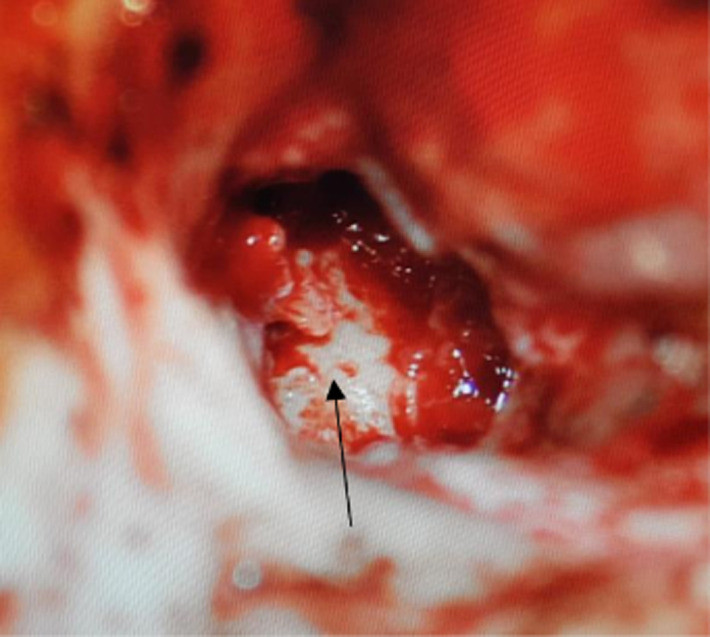
postoperative cavity after complete excision of the tumor

**Figure 7:**
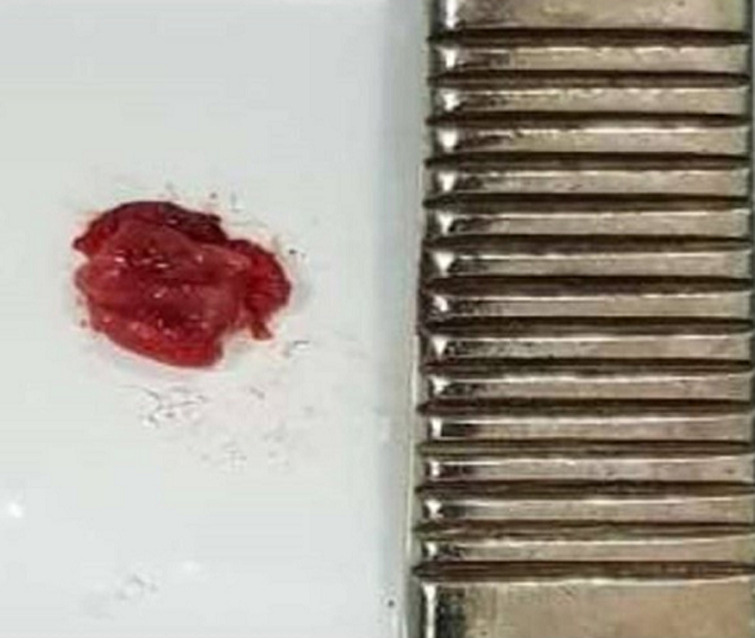
specimen: a un cm red mass

**Therapeutic interventions**: after considering the size of the tumor and its type, the decision was to proceed to a surgical treatment without primary embolization. The patient underwent a tympanotomy using a retro auricular access route. On per-operative examination, the tumor was hyper vascular, easily bleeding, emerging from the hypotympanum, filling the tympanic cavity and occluding the Eustachian tube (Figure 5). The intervention lasted two hours. The lesion was totally excised (Figure 6, Figure 7) without mastoidectomy which was judged unnecessary.

**Follow-up and outcome of interventions**: the postoperative course was uneventful. Anatomopathological microscopic examination revealed a benign tumor with cells arranged in nests and alveolar patterns separated by prominent blood vessels. Cells were with monomorphic fine granular chromatism nuclei and eosinophilic granular cytoplasm. Immunostaining for synaptophysin was positive and PS100 marked lenticular sus cells. The histopathologic findings thus confirmed diagnosis of paraganglioma. There was no recurrence during the one-year follow-up.

**Patient perspective**: the patient should share their perspective in one to two paragraphs on the treatment(s) they received.

**Informed consent**: the patient was informed by the different modalities of treatments and was consenting for surgery.

## Discussion

Head and neck paragangliomas (HNPs) represent neuroendocrine tumors developed from chromaffin cells within paraganglionic tissues of the autonomic nervous system [1]. They represent 0.03% of all human tumors and less than 0.5% of head-and-neck tumors. Their annual incidence is 0.001% [2,3]. Paraganglioma localizations vary widely. In Erickson´s series of 204 HNPs, 57% were localized in the carotid body, 30% were tympano-jugular and 13% vagal [4]. The gender ratio (F: M) is 2: 1. The average age of manifestation is the 5th decade [1]. Paragangliomas could occur sporadically or as hereditary tumors, especially in multiple endocrine neoplasia type II [1,5]. These forms appear in younger ages and present more multicentricity and bilateralism [1]. Pulsatile tinnitus is the most suggestive symptom of JTP. Progressive unilateral hearing loss could be caused by an ossicular lysis or a cochlear invasion. These signs were absent in our case. Less frequent manifestations are otalgia, vertigo, and neurological deficits (hoarseness, dysphagia and facial nerve palsy) [6]. Considering the slow growth rate of these tumors (0.8mm/year), an important consultation delay was reported [7]. Otoscopy reveals a retro-tympanic reddish-blue pulsatile mass [8], producing the “Rising Sun” appearance. Neurological examination may find Vernet syndrome secondary to invasion of the jugular foramen, with paralysis of the IX^th^, X^th^ and XI^th^ cranial nerves. Associated XII^th^ palsy forms Collet-Sicard syndrome [9]. On audiometry, a conductive hearing loss was found in all JTP, just like in our case. A sensorineural hearing loss could be associated in large tumors with intracranial extension [10]. Screening for catecholamines and their metabolites in urine and plasma in all cases of glomus tympanic tumor was previously reported. However, since catecholamine secretion occurs in only 1% to 4% of cases, recent studies recommend these investigations only in hereditary and multifocal forms, or in case of suggestive symptoms: diarrhea, flushing, hypertension and palpitations [11,12]. This explains why they were omitted in our case.

Imagery is necessary to diagnose HNP, classify the tumor and establish a treatment plan. Temporal bone CT and MRI are complementary [11]. Small JTP are seen on CT as well-defined intratympanic masses without significant bone involvement. Larger tumors can surround the ossicles, extend to the Eustachian tube, through the aditus to the mastoid and through the tympanic membrane to the external auditory canal. They could reach skull base neural foramina. CT scan has higher sensitivity to differentiate between tympanic glomus and jugular glomus by analyzing the bone plate separating the tympanic cavity from the jugular bulb. It could detect multicentric tumors along the vagus nerve and the carotid bifurcation [11]. The absence of bone destruction represents the most important differential feature with cholesteatoma. MRI is excellent to evaluate the soft tissue component of the tumor and its intracranial extension. The “salt-and-pepper” pattern of high and low signals on T2-weighted sequences and the contrast enhancement on T1-weighted sequences are nearly pathognomonic of paragangliomas [5,12]. In our case, brain MRI confirmed the diagnosis and classified it Type 2 of FISCH classification. Largely abandoned angiography could be indicated before embolization or if diagnosis is uncertain [11,12]. The 18F-dihydroxyphenylalanin positron emission tomography represents a promising diagnostic tool for smaller than 1cm paraganglioma´s [5]. Radiological examination allows tumor staging according to “FISCH classification” to establish adapted treatment modalities [11]. They include abstention, surgical resection, embolization and radiotherapy. Surgery remains the only radical treatment for JTPs. The surgical approach depends on the JTP´s localization and extension, it could be trans-canal or postauricular [7,12]. Preoperative embolization decreases bleeding during surgery. Post-operative complications are hearing loss, facial weakness, tympanic membrane perforation (1.7%), acquired cholesteatoma (1.7%), surgical-site infection (1.7%) and cerebrospinal fluid leak (0.9%) [12]. Jackson reports mortality rates of 0% to 4% [13].

Radiotherapy, used exclusively or postoperatively, could assure long-term tumor control. The recommended dose is 45-50 Gy fractioned over 5 weeks [7]. A study by Gilbo reported a local control of 96%, a cause-specific survival of 97% and an overall survival of 72% after a 10-year follow-up. No patient experienced severe complications [14]. Stereotactic radiotherapy treatment is an interesting alternative. It is administered in one fraction and applies minimal radiation to the adjacent normal tissues [7]. Close observation was described in literature as a possible strategy, with clinical and radiological evaluation every 6 to 12 months, extendable in case of stability [5,7,12,15]. It is considered in case of significant comorbidities, for elderly, or for pauci-symptomatic patients. A multidisciplinary consultation is useful to choose the most adapted treatment protocol. The patient´s clarified-consent is compulsory [7]. For Fisch class A and B, like our case, exclusive surgical resection is the gold standard. Fisch class C and D require postoperative radiotherapy. Embolization 24 to 48 hours before surgery is indicated for Fisch class B, C and D. As a palliative setting, it could be used to slow down tumor´s growth [5,7]. Ivan and Springate [16,17] describe tumor control rates of 86% after exclusive surgery, 69% after subtotal tumor resection, 71% to 90% after subtotal resection combined with adjuvant RT and 93% to 95% after exclusive RT. These findings go in pair with Miller´s conclusions [18,19]. In combined procedures, no recurrences were reported after a mean follow-up of 30.4 months [12].

## Conclusion

Jugular tympanic paraganglioma are challenging rare lesions. Precise clinical assessment, preoperative imaging and interdisciplinary discussions are the basis of an adequate management of HNPs. Function-preserving therapy seems to be the most adapted approach, even if, to date, there are still no consensual recommendations in literature.
